# Elemental Metabolomics and Pregnancy Outcomes

**DOI:** 10.3390/nu11010073

**Published:** 2019-01-02

**Authors:** Daniel R. McKeating, Joshua J. Fisher, Anthony V. Perkins

**Affiliations:** School of Medical Science, Menzies Health Institute Queensland, Griffith University, Southport 9726, Queensland, Australia; d.mckeating@griffith.edu.au (D.R.M.); josh.fisher@griffith.edu.au (J.J.F.)

**Keywords:** elemental metabolomics, trace elements, pregnancy, micronutrition

## Abstract

Trace elements are important for human health and development. The body requires specific micronutrients to function, with aberrant changes associated with a variety of negative health outcomes. Despite this evidence, the status and function of micronutrients during pregnancy are relatively unknown and more information is required to ensure that women receive optimal intakes for foetal development. Changes in trace element status have been associated with pregnancy complications such as gestational diabetes mellitus (GDM), pre-eclampsia (PE), intrauterine growth restriction (IUGR), and preterm birth. Measuring micronutrients with methodologies such as elemental metabolomics, which involves the simultaneous quantification and characterisation of multiple elements, could provide insight into gestational disorders. Identifying unique and subtle micronutrient changes may highlight associated proteins that are affected underpinning the pathophysiology of these complications, leading to new means of disease diagnosis. This review will provide a comprehensive summary of micronutrient status during pregnancy, and their associations with gestational disorders. Furthermore, it will also comment on the potential use of elemental metabolomics as a technique for disease characterisation and prediction.

## 1. Introduction

Biological trace elements are important for human health with imbalances in elemental homeostasis and metabolism playing a critical role in a variety of poor health outcomes. Micronutrition consists of elements required in small amounts in the daily diet that are essential for proper growth, development and physiology of organisms. For humans, this includes 13 elements that are not able to be synthesised, such as iron, selenium and calcium [1]. Recent studies suggest that, in some developed and relatively affluent societies, only 5% of the population meet the guidelines for adequate fruit and vegetable daily intake of 2 serves of fruit and 5 of vegetables, indicating that our diets may be lacking in essential nutrients that are principally acquired from these sources [2]. Maternal nutrition has long been considered to be important for a healthy pregnancy [3,4]. Adequate intake of macro nutrients has been correlated to positive pregnancy outcomes whereas hyperglycaemia, hyperlipidaemia and excessive calorific intakes have been associated with pregnancy complications. Similarly, the micronutrient status of women in many countries is below recommended daily intake (RDI) levels for both vitamins and minerals [5]. Due to the increasing demand for many micronutrients during pregnancy, the World Health Organization (WHO) recommends an increased intake of many nutrients during gestation and lactation [6]. Despite this, the global burden of maternal undernutrition including micronutrient deficiencies is persistent, particularly in South Asia where 10–40% are undernourished [7] Additionally, nutrients such as vitamin D, calcium, magnesium, and iron are consumed in quantities 74% lower than recommended levels in Australia; whilst vitamins A, C, and zinc consumption has been found to be 250% greater than recommendations, depending on the region [8].

Micronutrients play key roles in pregnancy outcomes, with aberrant micronutrition, such as deficiency in magnesium, potassium, calcium, selenium, and zinc being associated with poor perinatal outcomes such as gestational diabetes mellitus (GDM) and preeclampsia (PE), both are associated with an increase in other pregnancy complications including of fetal growth restriction (FGR), preterm birth and still birth [9,10]. Gestational disorders can lead to severe long term health outcomes for both mother and child after pregnancy, with this information in mind, it is of critical importance to identify women at risk of these complications as early in gestation as possible [11]. To limit the severity of negative outcomes or prevent disorders altogether, early detection and intervention is required. 

The use of elemental metabolomics; the study of elements present within an organism, has only recently been developed to a point which might be applicable to understanding human health. Recent studies have successfully utilised trace element metabolomics to predict the onset and progression of Alzheimer’s disease [12], Parkinson’s disease [13], diabetes [14], and cancer [15]. Multi-elemental analysis and predictive capabilities of this methodology could contribute to further understand gestational disorders and possible use as a means of predicting pregnancy outcomes. Complexities surrounding nutrition and pregnancy are extensive with various elements correlated to diverse outcomes. Currently, only a handful of essential elements are known to affect pregnancy outcomes, even though there may be additional micronutrients that are essential for pregnancy health and human development [16].

## 2. Maternal Nutrition

The inadequate levels of micronutrients are well understood in low income countries [17]; however, there is surprisingly little known about the micronutrient status of pregnant women in many developed countries. Women often supplement their diet with multiple micronutrients during pregnancy, and many more may consume the high-fat, low-nutrient diets typical of high-income nations. Various micronutrients are important for successful pregnancy, although the specific pregnancy related functions of many are poorly understood. Sedentary lifestyle, tobacco smoking, alcohol consumption, and hypertension are maternal risk factors that have been extensively shown to cause an increased incidence of negative outcomes during gestation. Micronutrients have roles in modulation of the maternal and fetal metabolism, oxidative stress, placentation, and structural development of key fetal organs and tissues [3,4]. 

The placenta plays a crucial role in mediating the transfer of nutrients via both active and passive transport mechanisms [18]. Previous research indicates that the placenta will preferentially uptake nutrients from the maternal system to prevent fetal deficiency [19,20]. Maternal conditions such as diabetes or obesity can alter the nutrient transporters in the placenta, leading to increased or decreased nutritional flow, with potential outcomes including overgrowth (macrosomia) or intrauterine growth restriction (IUGR) of the foetus. As the placenta coordinates many aspects of gestational development and maternal blood nourishes the foetus, biological samples from the maternal or fetal circulation provide highly meaningful information relating to micronutrient status, maternal health and fetal development. 

A concept popularised by David Barker known as “fetal programming” or the “Developmental Origins of Health and Disease” hypothesis, showed that there are a spectrum of processes that operate in all pregnancies and infancy that shape future health and risk of disease [21]. Fetal programming describes the maladaptive consequences to a maternal, fetal, or placental stressor during pregnancy that can result in abnormal development leading to disease [22]. 

Maternal nutrition has been specifically noted to have significant impacts on offspring outcome, as seen in the Dutch Famine (1944-45). After examining a cohort of 2414 people, it was noted that famine at any time during gestation was associated with glucose intolerance in offspring. If exposed early in gestation, there was an associated increase in obesity, coronary heart disease, atherogenic lipid profiles, disturbed blood coagulation, and increased stress responsiveness in offspring, while women who were conceived during this time had 5 times higher rates of breast cancer [23]. Although this study highlighted the complexities of human development, it also highlighted the importance of maternal nutrition and dietary profile of micro and macronutrition to the fetal environment. Though epidemiological studies have found correlations between over and under nutrition and programming of disease, few have managed to elucidate the mechanisms involved and the interrelationships of micronutrition in maternal and fetal health [24]. Currently, we only know how a selection of micronutrients affect pregnancy outcomes even though many additional micronutrients are likely to be essential to human development [16,25].

## 3. Micronutrition during Gestation and Lactation 

### 3.1. Potassium

The RDI of potassium during pregnancy is 2800 mg/day to avoid deficiency (hypokalaemia) (Table 1). Potassium can be acquired from sources such as leafy green, root vegetables, beans, peas and fruits. Meat products, nuts and dairy products also have moderate amounts of potassium [16]. Normal blood concentration of potassium ranges from 14.1 to 20.3 mg/dL, with levels lower than 9.7 mg/dL indicative of hypokalaemia [26]. During pregnancy, birth outcomes based around hypokalaemia are unknown, however there is the possibility of negative maternal outcomes such as extreme muscle fatigue [27] or muscular paralysis [28]. Hypokalaemia is also concomitant with cardiac arrhythmias and muscle weakness [29,30]. Hyperkalaemia (high potassium) is often used as a marker of acute metabolic or renal dysfunction and has also been associated with severe atherosclerotic morbidity leading to cardiovascular disease. Similar to hypokalaemia, there is limited literature associating hyperkalaemia and poor pregnancy health, although both GDM and PE patients have a higher possibility to develop renal dysfunction [31].

### 3.2. Calcium

Daily calcium intake during pregnancy is recommended at 1000 mg/day, increasing to 1200 mg/day in the last trimester (Table 1), with Vitamin D also consumed along with calcium to allow for the adaptive homeostatic mechanisms for gestation and lactation to occur [6,37]. In western diets, milk and milk products are the primary sources of calcium; with cereals, fruits, and vegetables making a lesser contribution. Calcium’s requirement by bone makes it critical during stages of rapid bone development, such as during gestation, infancy, childhood and adolescents [38]. Calcium is also important in the extracellular fluid for physiological function through mediation of cell signalling for both vasoconstriction, vasodilation, nerve transmission, contraction of muscles, and glandular secretion of hormones [38]. 

To maintain physiological functions within the body, calcium levels in the blood are maintained at around 8.6–10.2 mg/dL in adults, tightly controlled by the calcium sensing receptor, parathyroid hormone (PTH), and active vitamin D—1,25-Dihydroxyvitamin D [39]. Measurement of blood calcium is not an indication of total bone calcium, instead a representation of free calcium, a preferred indicator for those with protein abnormalities such as low albumin, which effects the ratio of free calcium vs. bound calcium. Measurements of calcium in urine are used to determine if renal excretion of calcium is normal. Serum concentration levels of calcium generally vary above or below the normal range under severe circumstances such as malnutrition or hyperparathyroidism [16]. When calcium levels decrease, the calcium sensing receptor triggers PTH release form the parathyroid gland which triggers the conversion of 25-hydroxyvitamin D to 1,25-Dihydroxyvitamin D, which increases circulating serum calcium levels from bone stores and increases smooth muscle contraction [39]. 

Low calcium levels are also concomitant with an increased release of renin from the kidneys, influencing maternal circulating renin-angiotensin-aldosterone systems (RAAS) leading to hypertension through vasoconstriction and fluid/sodium retention. The nonrenal renin-angiotensin systems (RAS) are important for ovulation, implantation, placentation, development of the uteroplacental, and umbilicoplacental circulation [40]. The function of the RAS in the maternal system is largely driven by maternal demand, and so the activity doesn’t reflect the role of RAS in the placenta which may have other documented roles in pathological pregnancies such as IUGR, and PE [40]. Calcium supplementation has been shown to be associated with a reduction in the risk of gestational hypertensive disorders and an increase in birthweight [41]. 

### 3.3. Magnesium

The recommended intake of magnesium during pregnancy is 350 mg/day to maintain function of over 300 enzymes that utilise ATP (Table 1) [6,16]. Leafy green vegetables provide a good source of magnesium, due to its presence in the core of the chlorophyll molecule. Other important sources include whole grains, nuts, legumes, cereals, and seafood. Water can also be an important source of magnesium, dependant on how “Hard” or “Soft” the water, in hard water communities it can comprise up to 38% of the daily magnesium consumption [42]. Enzymes in energy metabolism and neuromuscular signalling require magnesium for substrate formation. Whilst acting as an allosteric activator for phospholipase C, adenylate cyclase, and Na/K-ATPase; magnesium also regulates calcium ion transport channels, calcium homeostasis, and is required for calcium-triggered release of PTH and PTH action [43]. 

There is no generally accepted measurement for adequate magnesium status, as 50 to 60% resides within bone [16]. Serum magnesium is the best measurement available, but only measures <1% of total body magnesium. The normal reference range for serum is between 1.7 to 2.2 mg/dL, with levels below 1.7 mg/dL often referred to as hypomagnesaemia [16,44]. There is currently no literature surrounding how reference ranges for circulating magnesium should be altered during gestation. Magnesium deficiency is often accompanied by calcium and potassium deficiencies. Calcium becomes deficient due to the impaired PTH secretion caused by low magnesium, causing a perpetuation of low serum calcium. The rise is active vitamin D to recruit calcium from the gut that is expected to follow is attenuated under calcium deficient conditions, propagating calcium deficiency. With potassium, magnesium is required for it to be adequately conserved by the kidneys. When magnesium is low, potassium levels cannot be maintained and can proceed to hypokalaemia [43,44]. There may be a neuro protective effect of high maternal magnesium intake on offspring, whilst also having positive effects on bone mineral density [30,45].

### 3.4. Manganese

Involved in cellular metabolic processes, manganese is a component of antioxidant enzymes such as superoxide dismutase, and essential for development and human health. The bioavailability of manganese is low, primarily consumed through whole grains, nuts, seeds, and tea [16,45,46,47]. Uptake and retention is dictated by dietary calcium, iron, and phosphorus [48,49]. To reach an adequate intake of manganese during pregnancy, it is recommended that 5 mg/day be consumed (Table 1) [6]. Manganese is a cofactor for an extensive number of enzymes, including oxidoreductases, transferases, hydrolases, lyases, isomerases, ligases, lectins, and integrins [50]. Two of its more important roles however are arginase, the last enzyme for the urea cycle, and the mitochondrial antioxidant manganese super oxide dismutase [50]. 

Normal levels of manganese in the body range from 4–15 µg/L in blood, 0.4–0.85 µg/L in serum, and 1–8 µg/L in urine [46]. Serum, plasma and urine concentrations have all been noted to respond to dietary manganese intake, leading to disagreement on the best indicator of status [51,52]. During pregnancy, manganese concentrations have been shown to increase over the course of gestation from 10–34 weeks, however values fit within the currently established reference ranges [53,54]. Recent studies examining excessive manganese exposure due to environmental pollution have suggested that excessive manganese levels in pregnancy can negatively impact on the cognitive develop of the unborn child [55]. Despite this, there is still a considerable need to investigate the effects of manganese on foetal development during gestation [16].

### 3.5. Iron

The RDI for iron is 27 mg/day, however analysis of women living in America has found that 90% will not meet 22 mg/day (Table 1). Iron enters the diet through two different forms, haem and non-haem. Haem is found within meats, poultry and fish due to the iron component in the oxygen-transport metalloprotein haemoglobin [56]. Alternatively, non-haem iron is found in cereals, legumes, fruits, and vegetables. Non-haem forms make up a larger portion of the diet, however are not as bioavailable as haem forms [57]. Serum iron levels range from 50–170 µg/dL for women, pregnancy iron levels increase to keep up with the haemoglobin levels of both mother and child, however they appear to drop in concentration because of increased blood volume. There are no serum iron reference ranges for pregnant women, though blood haemoglobin levels have been shown to decrease from 12–16 g/dL in non-pregnant to 10–14 g/dL in pregnancy [16]. 

Iron is required in a wide range of enzymes and pathways for its redox properties and coordination chemistry [58]. In mammals, iron is integral to cellular respiration, oxygen transport, energy production, and DNA synthesis [59]. Knowledge surrounding disease associated with iron revolves around the understanding of iron homeostasis. Levels of iron may be affected by various factors such as genetic variations, dietary influence, absorption, and haemolysis. Iron deficiency is the main cause of anaemia, resulting from iron’s use in haemoglobin synthesis affecting healthy red blood cells [60]. High concentrations of iron within the blood can cause metabolic alterations resulting in increased incidence of insulin resistance and type 2 diabetes in pregnant women [30,61].

### 3.6. Copper 

The adequate intake of copper for pregnancy is 1.3 mg/day, with the upper safe intake being 8–10 mg/day (Table 1) [6]. Serum or plasma total copper levels are around 60–140 µg/dL for a healthy adult, with no adequate reference range for pregnant women [16]. Copper is an essential element for fundamental biological functions such as accepting and donating electrons in oxidation-reduction reactions, oxidative phosphorylation, free-radical detoxification, neurotransmitter synthesis, and iron metabolism [62]. Sources of foodstuffs with high levels of copper include meats, shellfish, nuts, and cocoa products. Foodstuffs low in copper can also provide a significant amount to a person’s intake, these include tea, potatoes, milk, and chicken [56]. 

Overnutrition of copper is mostly prevented during pregnancy by an emetic response, however can potentially result in toxic effects when over 15 mg are consumed [30]. Although there are no known associations with pregnancy overnutrition of copper, it has been considered a potential risk factor in cardiovascular disease [63,64]. High copper intake has also been negatively associated with cognitive function [65]. Undernutrition of copper is uncommon but can result from genetic uptake disorders or deficiency of trace elements from foodstuffs, more common in people consuming a Western diet [66]. Suboptimal intake in pregnancy may have negative effects on the developing lungs, skin, bones, organ systems and the immune system of the foetus. In newborns, copper deficiency manifests as oedema, anaemia, bone disease and recurrent apnoea [30,67]. Although copper is critical for pregnancy, supplementation is not recommended [16].

### 3.7. Zinc

Western diets meet the adequate daily intake of zinc for pregnant and lactating women of roughly 11 mg per day (Table 1) [16]. However, in analysis of upper and middle class households, it was found that greater than 30% of women did not reach this adequate intake level [68]. Zinc has both functional and structural roles in a number of enzyme systems that are important for gene expression, cell growth and division, neurotransmission, and reproductive and immune functions. A global search within the human genome has found that about 2800 proteins consist of a potential zinc binding site, making up around 10% of the proteome [69]. Shellfish, red meat, nuts, legumes, eggs, poultry, whole grains, some fruits and dairy products are all foodstuffs with a high bioavailability of zinc [16]. A meta-analysis by Foster, M. et al., (2015) on zinc status of vegetarians during pregnancy concluded that while vegetarian women have lower zinc intakes than non-vegetarian women, both groups consume lower than recommended amounts [70]. As a method of zinc status diagnosis, serum and plasma are used as a marker of deficiency with normal serum zinc levels ranging for 0.66–1.10 µg/mL [16].

Imbalances in zinc homeostasis are associated with a variety of human diseases. Often accompanied by malnourishment, zinc deficiency is estimated at 20–30% of the global population. Zinc is essential to physical and neurological development of infants and children, whilst also vital to protect plasma membranes from oxidative damage [71]. Immune system function is affected by zinc deficiency, increasing the risk and severity of infection [72]. Zinc also plays a significant role in neurological development of infants and children, with deficiency associated with neuronal atrophy, behavioural problems, and impaired cognitive development [16]. A number of poor gestational outcomes are associated with inadequate zinc intake. Zinc overnutrition is primarily associated with maternal gastrointestinal stress which has not been associated with poor maternal outcomes or fetal programming [16,30].

### 3.8. Iodine

The RDI for iodine is 220 µg/day during pregnancy (Table 1), however according to the WHO there is concern for iodine availability in areas around Europe, the Eastern Mediterranean, Africa, Himalayas, Andes, and Western Pacific [6,73]. Iodine is a key component of thyroid hormones, thyroxine (T4) and triiodothyronine (T3) which regulate growth, development, reproductive function, metabolic rate, cellular metabolism, and connective tissue integrity [74]. All biological actions of iodine are attributed to thyroid hormones. Seafoods and some dairy foodstuffs contain a large concentration of iodine. Whilst most iodine is derived from these sources, it is also possible to consume an adequate amount of iodine through eggs, meats, and bread [56]. Iodine is primarily excreted through urine and as a result is a good indicator of dietary iodine intake, with sufficiency defined as 150–249 µg/L [75]. 

Low iodine is correlated to impaired neurological development, in particular neuropsychological leading to cretinism, mental retardation and brain damage [30,76]. Attention Deficit Hyperactivity Disorder is also more common in offspring, this is believed to be due to the disruption of brain development and myelination of the central nervous system in utero [77]. Excess iodine intake has its own accompanying risks. Symptoms of acute toxicity involve diarrhoea, hyperactivity, weakness, convulsions, and possibly death [78]. Women with excessive iodine intakes are found to be more likely to be suffering from thyroid related diseases such as hypothyroidism resulting in maternal weight gain and haemolysis possibly resulting in negative fetal outcomes and death [79].

### 3.9. Selenium

Selenium is an essential trace element required in small amounts in the diet to comprise the primary component of selenoproteins that have various roles including antioxidant function [80]. It is recommended during pregnancy that women have an intake of 60 µg/day of selenium [81]. Food sources of selenium are affected by the soil content in which they are grown so in areas with proficient selenium in the soil there is a greater intake. Also, areas with high levels of sulphur in the soil are known to have significantly reduced concentrations of selenium in the diet due to the competitive absorption of sulphur over selenium [82]. Brazil nuts are particularly high in organic selenium content and can lead to over nutrition if consumed in substantial amounts [83]. Selenium can also be obtained from cereals and a variety of fruits and vegetables, with 30–40% of dietary intake found in meats, fish and poultry [84]. There are a number of markers of selenium concentrations in the body [85]. Urinary excretion of selenium should range between 15–50 µg/L and is a marker of intake status. Selenium status can also be measured within the blood serum where the reference range is between 70–150 ng/mL for adults and 45–90 ng/mL for newborns [16].

Selenium exerts its functions in the body in the form of selenocysteine (SeCys), the 21st amino acid in the body [86]. Selenoproteins often contain SeCys, the main selenoprotein families are the thioredoxin reductases (TRxR), glutathione peroxidases (GPx), and iodothyronine deiodinases (IDO) [87]. Both TRxR and GPx are antioxidants that protect the body from high levels of oxidative stress; whereas the IDO’s are used to convert the inactive thyroid hormone, T4, to the biologically active form, T3 [88]. The thyroid is thus sensitive to selenium concentrations, and deficiency can result in an exacerbation of iodine deficiency [88]. As discussed subsequently, selenium deficiency during gestation has been associated with a number of negative outcomes, these include miscarriages, premature birth, low birth weight, and preeclampsia [36]. Consistently high levels of selenium intake can lead to selenosis: symptoms include gastrointestinal distress, hair loss, brittle fingernails and fatigue. Selenosis has also been shown to cause mild nerve damage and increase the risk of type-2 diabetes mellitus. The effects of overnutrition of selenium on offspring is unknown, however it may reduce the risk of maternal hyperthyroidism and lymphocytic thyroiditis [76]. 

## 4. Micronutrition in Gestational Complications

Poor micronutrition is often concomitant with increased incidence of gestational disorders such as GDM, PE, IUGR, and preterm pregnancies [10,11,16]. The literature surrounding micronutrition is also very complex, highlighting interactions that occur between various elements in physiological systems, and is limited with regards to heavy metals that are not classified as essential micronutrients.

### 4.1. Gestational Diabetes Mellitus

Gestational diabetes mellitus is a severe complication of pregnancy with an increasing prevalence, doubling incidence over an 8-year period [89,90]. The syndrome results from an impaired capacity of maternal beta cells to adapt to decreased insulin sensitivity that occurs during gestation, impairing glucose tolerance during pregnancy. The subsequent increased glucose levels that accompany GDM can further impair the development of the placenta and fetal growth [91]. In addition, maternal metabolic effects of GDM increase the chance of weight issues and poor pancreatic function. The effects on the foetus include increased adiposity and fetal hyperinsulinemia [92]. Poorly managed cases of GDM have been shown to result in increased cases of hypoglycaemia, primary caesarean deliveries, and large for gestational age offspring [93]. Although GDM can also cause IUGR, it is more likely to result in macrosomic foetuses [91].

Low levels of circulating potassium have been associated with impaired glucose tolerance due to the reduced ability for the pancreas to secret insulin [92]. Low selenium and chromium intake have also been associated with GDM [94]. Low serum concentrations and urinary excretion of zinc have been shown to be associated with diabetes [95] and zinc efficiency is also known to reduce growth factor signalling, particularly the insulin-like growth factor axis [96,97]. A review by Zhang, C. and Rawal, S. (2017) systematically evaluated the effects of iron intake, and iron status in GDM suggesting that a potential link between greater iron stores or status during gestation and an increased risk of GDM [98].

### 4.2. Pre-eclampsia

Occurring in approximately 3–5% of pregnancies, PE is associated with over 60,000 maternal deaths a year and increases perinatal mortality 5-fold [99]. Characterised by maternal endothelial cell dysfunction, resulting in symptoms that include maternal hypertension and proteinuria in late gestation; there is no current early means of detection for PE. Believed to originate from abnormal implantation and vascular development of the placenta, resulting in placental dysfunction, the initial cause of PE is poor uterine and placental perfusion, which can lead to hypoxic conditions and increased oxidative stress [100]. In some cases, the condition may progress to eclampsia, leading to seizures, coma, and ultimately death [101]. 

Hypertension exhibited with PE is linked to the sodium:potassium ratio. Blood pressure is correlated directly with sodium intake and inversely with potassium; however, potassium intake has not been associated with hypertensive disorders in pregnancy [102]. A systematic review of 13 studies on calcium supplementation during pregnancy found that with supplementation of 1 g/day, the average risk of PE was reduced by 55%, whilst gestational hypertensive disorders were reduced by 35% [103]. Due to magnesium deficiency being concomitant to calcium and potassium levels, it has been linked to PE. Magnesium levels in women with PE have been shown to be significantly reduced when compared to normal pregnant controls [46,104]. The decreased function of antioxidants that is accompanied with selenium and zinc deficiency has led to them being associated with preeclampsia, known to be correlated with high levels of oxidative stress [105].

### 4.3. Intrauterine Growth Restriction

Fetal growth restriction or IUGR occurs when the foetus fails to reach its expected growth potential at the appropriate gestational age. Associated with increased perinatal morbidity and mortality, IUGR is responsible for 30% of stillbirths, and increased incidence of premature births [106]. While IUGR babies may be born small for gestational age (SGA), IUGR babies are more likely to show signs of placental disease and have worse perinatal outcomes than SGA babies [107]. IUGR is mostly caused by the placenta through either poor placental function and/or insufficiency and a failure to adapt to improve fetal growth [108,109,110], this is known to be associated with an increase in diseases throughout life [111]. 

Reduced uterine blood flow may occur due to maternal hypotension or renal disease, which can lead to a reduced nutrient transport to the foetus which can cause IUGR [112,113]. Studies suggest that lower maternal manganese levels are associated with IUGR and an increased incidence of lower birth weight [114], whilst iron over nutrition has been linked to an increased possibility for SGA babies [16]. During pregnancy, a poor intake of zinc is teratogenic, causing IUGR and structural abnormalities [30,115]. Iodine deficiency is associated with a number of negative outcomes. Areas deficient in iodine see an increase in both birthweight and head circumference in offspring [116]. Low selenium intake has also been associated with IUGR, and recurrent miscarriage [32].

### 4.4. Preterm Birth

It’s estimated 1:10 babies are born prematurely with approximately one million children dying each year due to complications associated with preterm birth. Factors such as maternal stress and inflammation have been associated, however literature suggests that placental ischemia or other forms of placental dysfunction are more likely to contribute to preterm birth [117]. There are higher rates of disability in children and increased risk of disease susceptibility throughout life with preterm births, with the chances increased the earlier a child is born [118,119].

Pre-term delivery was shown to be reduced in women supplemented with calcium. A WHO randomised control trial of >8000 women with <600 mg/day calcium intake showed that those with calcium supplementation had statistically lower incidence of pre-term birth than the placebo group (2.6% supplemented vs. 3.2% placebo) [120]. Low magnesium levels have also been correlated with increased incidence of pre-term birth and low birth weight in offspring in humans [45]. Iron deficiency has been associated with poor growth development, premature birth, and impairment of cognitive skills and neurodevelopment [16]. Women with low haemoglobin levels have been found to have double the risk of premature delivery and low birthweight in offspring [121,122]. Two studies have correlated low selenium concentrations in maternal and cord blood to pre-term birth, with evidence suggesting that adequate maternal selenium may protect against pre-term deliveries [123,124].

## 5. Profiling Micronutrients in Disease

The complexities surrounding micronutrition during pregnancy are profound. As previously stated, over and under nutrition of various elements can be correlated to diverse outcomes. Therefore, measuring a small number of variables in isolation may not be able to give an accurate representation of disease or be able to monitor disease progression. By measuring the pattern of many trace elements, various disease conditions may be better characterised and therefore treated. Early work using elemental metabolomics for Alzheimer’s disease, Parkinson’s, type 2 diabetes, and cancer has yielded promising results for both characterisation and understanding of disease.

### Elemental Metabolomics

Elemental metabolomics (also known as ionomics), is a technique which involves the simultaneous quantification and characterisation of many chemical elements with high-throughput elemental analysis technologies and their integration with bioinformatics tools [125]. The concepts behind elemental metabolomics were established in the early 1940s, introducing terms like “metabolic patterns”, “individual metabolic patterns”, and “hypothetical average individual”. It is only with recent advances in sensitivity, throughput, instrumentation, cheminformatics, and bioinformatics that determining the detailed metabolic pattern of an individual has become feasible [126]. This allows the establishment of “elemental signature which may prove to be powerful diagnostic aids.

**Figure 1:**
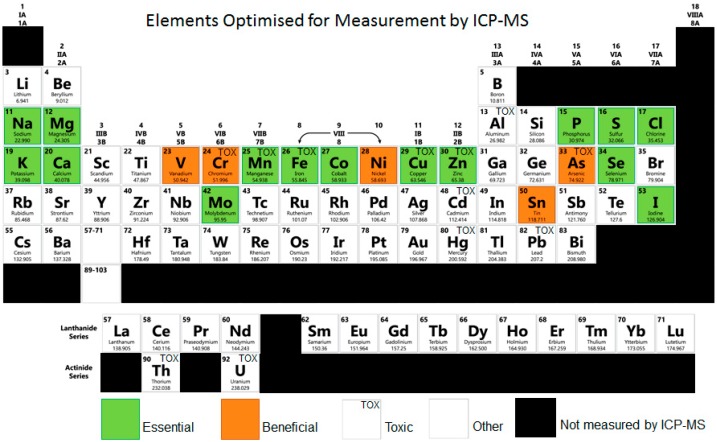
Elements measurable by ICP-MS, image adapted from Zhang, P. et al., (2017) [126]. Classified into essential, beneficial, others without a clearly defined function, and toxic elements. Essential and beneficial elements comprise major structural components (Ca, P, S); components of hormones or enzymes (Co, Cr, Cu, Fe, I, Mn, Mo, Ni, Se, Sn, V, Zn); responsible for maintenance of ionic equilibria, activation, or signalling (Ca, K, Mg, Na).

The majority of techniques used in elemental analysis include inductively coupled plasma mass spectrometry (ICP-MS), inductively coupled plasma optical emission spectroscopy (ICP-OES), and X-ray fluorescence [127]. Among them, ICP-MS is the most frequently used approach, which is capable of detecting metals as low as parts per trillion. Compared to other methodologies, ICP-MS allows for smaller sample size owing to its greater sensitivity and has the ability to detect different isotopes of the same element (Figure 1) Currently, ICP-MS has been successfully used for large scale elemental studies in yeast, plants and mammals [128,129]. These investigations illustrate the power of metabolomics to identify new aspects of trace element metabolism and homeostasis, and how such information can be used to develop hypotheses regarding the functions of previously uncharacterised diseases [130,131].

ICP-MS has been used for the quantification of elements in a variety of different human diseases, but the pattern of elements in disease states are far less well categorised. Even so, the limited work that has been conducted in the medical field has yielded promising results for various conditions such as Alzheimer’s disease, Parkinson’s, type 2 diabetes, and cancer. Little work has been conducted on the determination of elemental metabolomic profiles during gestation. Once established, elemental metabolomic profiles could better characterise various biological conditions and pathologies, providing novel treatment options. For example, recently it has been shown that metabolomics can identify genes and gene networks that directly control the metabolome [131]. In addition, this technique may provide a powerful tool for investigating more complex networks in gestation that control developmental and physiological processes that influence the metabolome indirectly. 

With the accelerated development of elemental metabolomics, advanced strategies have been developed for systematic analysis of chemical elements. A recent study identified a distinctive pattern of serum elements during the progression of Alzheimer’s disease. With the metabolomic analysis of elemental ratios, this study was able to differentiate with 90% accuracy between diseased and healthy individuals. Essential elements such as manganese, selenium, zinc and iron were shown to increase initially with early onset of Alzheimer’s then decrease with the development of mild cognitive impairment and ultimately true Alzheimer’s disease [12]. 

In another study, trace elements in plasma were analysed for 238 diagnosed Parkinson’s patients and 302 controls. Their findings indicated that lower plasma selenium and iron concentrations may reduce the risk of developing the disease, whereas lower plasma zinc was associated with an increased risk factor. They also used a model to then predict patient disease status based on several trace elements, also defining other features such as sex and age, highlighting possibilities for future computational strategies to improve elemental metabolomic studies [13].

Sun et al., analysed the fasting elemental concentrations of 976 middle-aged Chinese men and women to determine associations of ion modules and networks with obesity, metabolic syndrome, and type 2 diabetes mellitus (T2D). They found that copper and phosphorus were always ranked as the first two specific ion networks in people with health complications, whilst specific elemental patterns were also observed for each of the conditions [132]. Another study noted that increased urinary nickel concentrations were associated with an increased incidence of T2D in 2115 Chinese aged 55–76 years old [14].

Golasik et al., investigated the relationship between elemental status and cancer risk to support diagnosis. Disturbance in the homeostasis of metals is among one of many factors that can cause cancer malignancy. Through analysing both essential elements (calcium, magnesium, zinc, manganese, copper, iron etc.) and toxic elements (cadmium and lead) in hair and nail samples of patients with laryngeal cancer, they noted that most of the essential elements were significantly reduced in cancer patients, whilst toxic elements increased. Using a variety of bioinformatic techniques they were also able to determine classifiers for prediction of cancer probability, which may be useful for estimating risk, and early screening of cancer [15]. 

Elemental studies have also been used to examine metal concentrations in saliva and blood of periodontal disease patients. Using cluster analysis of metals in the classifications of samples, the resulting clusters suggested the elemental profiles of those with periodontal disease are different from controls. These researchers concluded that this may become a basis for future diagnostic and prognostic tools for periodontal disease [133]. 

## 6. Conclusions

Gestational complications such as GDM, PE, IUGR, and preterm birth can have lifelong consequences for the health of the mother and child. However, pregnancy disorders such as these are poorly understood despite extensive research. Little work has been conducted on the determination of elemental metabolomic profiles during gestation and how this might be influenced by maternal nutrition. Once established, elemental metabolomic profiles could better characterise various biological conditions and pathologies, providing novel treatment options. Currently, we only know how a select few micronutrients affect pregnancy outcomes [25], even though many additional micronutrients are likely to be essential to human development. 

Development of elemental metabolomic technology for generating a micronutrient “signature” or “fingerprint” as a predictive biomarker of pregnancy complications could provide vital information on relationships between specific micronutrients and pregnancy complications. Many key proteins in the body require specific micronutrients to function, therefore screening to identify novel and subtle micronutrient changes could highlight associated proteins that may affected and underpinning the pathophysiology of these complications. This would open up the way for simple interventions and therapies which could prove to be of immense benefit to both mother and baby. 

## Figures and Tables

**Table 1:** Recommended daily intake (RDI) and reference ranges for elements. Information gathered from Nutrient Reference Values for Australia and New Zealand. New Zealand Ministry of Health, 2006. [8], Gluckman, P., et al., Nutrition and lifestyle for pregnancy and breastfeeding. 2014: Oxford University Press, UK. [16], Farinde, A. Lab Values, Normal Adult: Laboratory Reference Ranges in Healthy Adults. Available online: https://emedicine.medscape.com/article/2172316-overview. [32], FSANZ. The 23rd Australian total diet study; Food Standards Australia New Zealand: 2011. [33]. Additional information from [34,35]. Elements that are not present did not have documented reference ranges. This is due to their normal concentration being unknown, or they are not in measurable quantities in human blood, serum, plasma, urine, or cord blood.

	RDI	Blood Reference Range	Serum/Plasma Reference Range	Urine Reference Range	Cord Reference Range
Na (Sodium)	460–920 mg [6]	310–335 mg/dL [36]	-	45 mg/dL [36]	290–380 mg/dL [36]
Mg (Magnesium)	350 mg [6]	3.6–6.1 mg/dL [36]	1.7–2.2 mg/dL [16]	145–245 mg/day [36]	-
P (Phosphorus)	1000 mg [6]	6–9.2 mg/dL [36]	2.7–4.4 mg/dL [36]	0.4–1.3 g/day [36]	-
S (Sulfur)	900 mg [6]	-	-	-	-
K (Potassium)	2800 mg [6]	14.1–20.3 mg/dL [36]	13.3–17.2 mg/dL [36]	97.7–490 mg/dL [36]	21.9–46.92 mg/dL [36]
Ca (Calcium)	1000 mg [6]	8.6–10.2 mg/dL [36]	8.6–10 mg/dL [36]	100–300mg/day [36]	-
V (Vanadium)	<1.8 mg [32]	-	<1 µg/L [36]	-	-
Cr (Chromium)	30 µg [6]	0.5–2.5 µg/L [33]	0.8–5.1 µg/mL [33]	-	-
Mn (Manganese)	5 mg [6]	4–15 µg/L [36]	0.4–0.85 µg/L [36]	1–8 µg/L [36]	-
Fe (Iron)	27 mg [6]	-	50–170 µg/dL [36]	-	-
Co (Cobalt)	-	0.7–3.4 µg/L [33]	0.3–7.5 µg/L [33]	-	-
Ni (Nickel)	-	-	<2 µg/L [36]	-	-
Cu (Copper)	1.3 mg [6]	70–140 µg/dL [36]	80–155 µg/dL [36]	3–35 µg/day [36]	4.6–8.8 µmol/L [34]
Zn (Zinc)	11 mg [6]	4.5–6.5 mg/L [36]	0.66–1.10 µg/mL [36]	-	15.8–22 µmol/L [34]
As (arsenic)	-	0.2–2.3 µg/dL [36]	-	5–50 µg/day [36]	-
Se (Selenium)	65 µg [6]	-	70–150 µg/L [36]	15–50 µg/L [36]	0.5–0.7 µmol/L [34]
Mo (Molybdenum)	50 µg [16]	0.6–4 µg/L [36]	0.3–2.0 µg/L [36]	-	-
I (Iodine)	220 µg [6]	-	40–92 µg/L [36]	150–249 µg/L [36]	-

## References

[1] Mertz W. (1981). The essential trace elements. Science.

[2] Australian Bureau of Statistics: A.B.O National Health Survey: First Results, 2014-15. http://www.abs.gov.au/AUSSTATS/abs@.nsf/DetailsPage/4364.0.55.0012014-15?OpenDocument.

[3] Abu-Saad K., Fraser D. (2010). Maternal nutrition and birth outcomes. Epidemiol. Rev..

[4] Blumfield M.L., Hure A.J., Macdonald-Wicks L., Smith R., Collins C.E. (2013). A systematic review and meta-analysis of micronutrient intakes during pregnancy in developed countries. Nutr. Rev..

[5] Darnton-Hill I., Mkparu U.C. (2015). Micronutrients in pregnancy in low- and middle-income countries. Nutrients.

[6] WHO/FAO (2004). Vitamin and Mineral Requirements in Human Nutrition.

[7] Ahmed T., Hossain M., Sanin K.I. (2012). Global burden of maternal and child undernutrition and micronutrient deficiencies. Ann. Nutr. Metab..

[8] New Zealand Ministry of Health Nutrient Reference Values for Australia and New Zealand. https://www.health.govt.nz/publication/nutrient-reference-values-australia-and-new-zealand.

[9] Vanderlelie J., Scott R., Shibl R., Lewkowicz J., Perkins A., Scuffham P.A. (2016). First trimester multivitamin/mineral use is associated with reduced risk of pre-eclampsia among overweight and obese women. Matern. Child Nutr..

[10] Gernand A.D., Schulze K.J., Stewart C.P., West K.P., Christian P. (2016). Micronutrient deficiencies in pregnancy worldwide: Health effects and prevention. Nat. Rev. Endocrinol..

[11] Cheong J.N., Wlodek M.E., Moritz K.M., Cuffe J.S. (2016). Programming of maternal and offspring disease: Impact of growth restriction, fetal sex and transmission across generations. J. Physiol..

[12] Paglia G., Miedico O., Cristofano A., Vitale M., Angiolillo A., Chiaravalle A.E., Corso G., Di Costanzo A. (2016). Distinctive pattern of serum elements during the progression of Alzheimer’s disease. Sci. Rep..

[13] Zhao H.-W., Lin J., Wang X.-B., Cheng X., Wang J.-Y., Hu B.-L., Zhang Y., Zhang X., Zhu J.-H. (2013). Assessing plasma levels of selenium, copper, iron and zinc in patients of Parkinson’s disease. PLoS ONE.

[14] Liu G., Sun L., Pan A., Zhu M., Li Z., Wang Z., Liu X., Ye X., Li H., Zheng H. (2014). Nickel exposure is associated with the prevalence of type 2 diabetes in Chinese adults. Int. J. Epidemiol..

[15] Golasik M., Jawień W., Przybyłowicz A., Szyfter W., Herman M., Golusiński W., Florek E., Piekoszewski W. (2015). Classification models based on the level of metals in hair and nails of laryngeal cancer patients: Diagnosis support or rather speculation?. Metallomics.

[16] Gluckman P., Hanson M., Seng C.Y., Bardsley A. (2014). Nutrition and Lifestyle for Pregnancy and Breastfeeding.

[17] Fall C.H., Fisher D.J., Osmond C., Margetts B.M. (2009). Multiple micronutrient supplementation during pregnancy in low-income countries: A meta-analysis of effects on birth size and length of gestation. Food Nutr. Bull..

[18] Gaccioli F., Lager S. (2016). Placental nutrient transport and intrauterine growth restriction. Front. Physiol..

[19] Antony A.C. (1996). Folate receptors. Annu.Rev. Nutr..

[20] Solanky N., Jimenez A.R., D’Souza S., Sibley C., Glazier J. (2010). Expression of folate transporters in human placenta and implications for homocysteine metabolism. Placenta.

[21] Barker D.J., Osmond C. (1986). Infant mortality, childhood nutrition, and ischaemic heart disease in England and Wales. Lancet.

[22] Thornburg K.L., Shannon J., Thuillier P., Turker M.S. (2010). In utero life and epigenetic predisposition for disease. Adv. Genet..

[23] Roseboom T.J., Van Der Meulen J.H., Ravelli A.C., Osmond C., Barker D.J., Bleker O.P. (2001). Effects of prenatal exposure to the Dutch famine on adult disease in later life: An overview. Mol. Cell. Endocrinol..

[24] Grieger J.A., Clifton V.L. (2014). A review of the impact of dietary intakes in human pregnancy on infant birthweight. Nutrients.

[25] Lewicka I., Kocyłowski R., Grzesiak M., Gaj Z., Oszukowski P., Suliburska J. (2017). Selected trace elements concentrations in pregnancy and their possible role—Literature review. Ginekol. Pol..

[26] Burtis C.A., Ashwood E.R., Bruns D.E. (2006). Tietz Textbook of Clinical Chemistry and Molecular Diagnostics.

[27] Matsunami K., Imai A., Tamaya T. (1994). Hypokalemia in a pregnant woman with long-term heavy cola consumption. Int. J. Gynecol. Obstet..

[28] Appel C.C., Myles T.D. (2001). Caffeine-induced hypokalemic paralysis in pregnancy. Obstet. Gynecol..

[29] Khaw K., Barrett-Connor E. (1988). The association between blood pressure, age, and dietary sodium and potassium: A population study. Circulation.

[30] Hofstee P., McKeating D.R., Perkins A.V., Cuffe J.S. (2018). Placental adaptations to micronutrient dysregulation in the programming of chronic disease. Clin. Exp. Pharmacol. Physiol..

[31] Wolak T., Shoham-Vardi I., Sergienko R., Sheiner E. (2016). High potassium level during pregnancy is associated with future cardiovascular morbidity. J. Matern. Fetal Neonatal Med..

[32] Farinde A. Lab Values, Normal Adult: Laboratory Reference Ranges in Healthy Adults. https://emedicine.medscape.com/article/2172316-overview.

[33] FSANZ (2011). The 23rd Australian Total Diet Study.

[34] Jantzen C., Jørgensen H.L., Duus B.R., Sporring S.L., Lauritzen J.B. (2013). Chromium and cobalt ion concentrations in blood and serum following various types of metal-on-metal hip arthroplasties: A literature overview. Acta Orthop..

[35] Galinier A., Périquet B., Lambert W., Garcia J., Assouline C., Rolland M., Thouvenot J.-P. (2005). Reference range for micronutrients and nutritional marker proteins in cord blood of neonates appropriated for gestational ages. Early Human Dev..

[36] Mariath A.B., Bergamaschi D.P., Rondó P.H., Ana C.A.T., de Fragas Hinnig P., Abbade J.F., Diniz S.G. (2011). The possible role of selenium status in adverse pregnancy outcomes. Br. J. Nutr..

[37] Brown E.M. (2011). Vitamin D and the Calcium-Sensing Receptor. Vitamin D.

[38] Brini M., Ottolini D., Calì T., Carafoli E., Sigel A., Sigel H., Sigel R. (2013). Calcium in health and disease. Interrelations between Essential Metal Ions and Human Diseases.

[39] Jorde R., Sundsfjord J., Haug E., Bønaa K.H. (2000). Relation between low calcium intake, parathyroid hormone, and blood pressure. Hypertension.

[40] Lumbers E.R., Pringle K.G. (2013). Roles of the circulating renin-angiotensin-aldosterone system in human pregnancy. Am. J. Physiol. Regul. Integr. Comp. Physiol..

[41] Imdad A., Bhutta Z.A. (2012). Effects of calcium supplementation during pregnancy on maternal, fetal and birth outcomes. Paediatr. Perinat. Epidemiol..

[42] Fatemi S., Ryzen E., Flores J., Endres D.B., Rude R.K. (1991). Effect of experimental human magnesium depletion on parathyroid hormone secretion and 1,25-dihydroxyvitamin D metabolism. J. Clin. Endocrinol. Metab..

[43] Arnaud M.J. (2008). Update on the assessment of magnesium status. Br. J. Nutr..

[44] Rude R.K. (1998). Magnesium deficiency: A cause of heterogenous disease in humans. J. Bone Miner. Res..

[45] Spencer B., Vanderlelie J., Perkins A. (2015). Essentiality of trace element micronutrition in human pregnancy: A systematic review. J. Pregnancy Child Health.

[46] Williams M., Todd G., Roney N., Crawford J., Coles C., McClure P., Garey J., Zaccaria K., Citra M. (2012). Toxicological Profile for Manganese.

[47] Fitsanakis V.A., Zhang N., Garcia S., Aschner M. (2010). Manganese (Mn) and iron (Fe): Interdependency of transport and regulation. Neurotox. Res..

[48] Greger J. (1998). Dietary standards for manganese: Overlap between nutritional and toxicological studies. J. Nutr..

[49] Freeland-Graves J.H., Lin P.-H. (1991). Plasma uptake of manganese as affected by oral loads of manganese, calcium, milk, phosphorus, copper, and zinc. J. Am. Coll. Nutr..

[50] Law N.A., Caudle M.T., Pecoraro V.L. (1998). Manganese redox enzymes and model systems: Properties, structures, and reactivity. Adv. Inorg. Chem..

[51] Davis C.D., Greger J. (1992). Longitudinal changes of manganese-dependent superoxide dismutase and other indexes of manganese and iron status in women. Am. J. Clin. Nutr..

[52] Friedman B., Freeland-Graves J.H., Bales C.W., Behmardi F., Shorey-Kutschke R.L., Willis R.A., Crosby J.B., Trickett P.C., Houston S.D. (1987). Manganese balance and clinical observations in young men fed a manganese-deficient diet. J. Nutr..

[53] Tholin K., Sandström B., Palm R., Hallmans G. (1995). Changes in blood manganese levels during pregnancy in iron supplemented and non supplemented women. J. Trace Elem. Med. Biol..

[54] Spencer A. (1999). Whole blood manganese levels in pregnancy and the neonate. Nutrition.

[55] Henn B.C., Bellinger D.C., Hopkins M.R., Coull B.A., Ettinger A.S., Jim R., Hatley E., Christiani D.C., Wright R.O. (2017). Maternal and cord blood manganese concentrations and early childhood neurodevelopment among residents near a mining-impacted superfund site. Environ. Health Perspect..

[56] Institute of Medicine Dietary (2001). Vitamin A, Vitamin K, Arsenic, Boron, Chromium, Copper, Iodine, Iron, Manganese, Molybdenum, Nickel, Silicon, Vanadium, and Zinc.

[57] Hulten L., Gramatkovski E., Gleerup A., Hallberg L. (1995). Iron absorption from the whole diet. Relation to meal composition, iron requirements and iron stores. Eur. J. Clin. Nutr..

[58] Hider R.C., Kong X. (2013). Iron: Effect of overload and deficiency. Met. Ions Life Sci..

[59] Henderson B., Kühn L. (1997). Interaction between iron-regulatory proteins and their RNA target sequences, iron-responsive elements. Prog. Mol. Subcell. Biol..

[60] Koulaouzidis A., Said E., Cottier R., Saeed A.A. (2009). Soluble transferrin receptors and iron deficiency, a step beyond ferritin. A systematic review. J. Gastrointest. Liver Dis..

[61] Dandona P., Hussain M., Varghese Z., Politis D., Flynn D., Hoffbrand A. (1983). Insulin resistance and iron overload. Ann. Clin. Biochem..

[62] Peña M.M., Lee J., Thiele D.J. (1999). A delicate balance: Homeostatic control of copper uptake and distribution. J. Nutr..

[63] McKeown N.M. (2004). Whole grain intake and insulin sensitivity: Evidence from observational studies. Nutr. Rev..

[64] Trumbo P., Schlicker S., Yates A.A., Poos M. (2002). Dietary reference intakes for energy, carbohydrate, fiber, fat, fatty acids, cholesterol, protein and amino acids. J. Am. Diet. Assoc..

[65] Drehmer M., Camey S.A., Nunes M.A., Duncan B.B., Lacerda M., Pinheiro A.P., Schmidt M.I. (2013). Fibre intake and evolution of BMI: From pre-pregnancy to postpartum. Public Health Nutr..

[66] Klevay L.M. (2011). Is the Western diet adequate in copper?. J. Trace Elem. Med. Biol..

[67] Al-Rashid R.A., Spangler J. (1971). Neonatal copper deficiency. N. Engl. J. Med..

[68] Prasad A.S. (2014). Impact of the discovery of human zinc deficiency on health. J. Trace Elem. Med. Biol..

[69] Andreini C., Banci L., Bertini I., Rosato A. (2006). Counting the zinc-proteins encoded in the human genome. J. Proteome Res..

[70] Foster M., Herulah U.N., Prasad A., Petocz P., Samman S. (2015). Zinc status of vegetarians during pregnancy: A systematic review of observational studies and meta-analysis of zinc intake. Nutrients.

[71] O’Dell B.L. (2000). Role of zinc in plasma membrane function. J. Nutr..

[72] Mossad S.B., Macknin M.L., Mendendorp S.V., Mason P. (1996). Zinc gluconate lozenges for treating the common cold: A randomized, double-blind, placebo-controlled study. Ann. Intern. Med..

[73] Andersson M., De Benoist B., Darnton-Hill I., Delange F.M., Organization W.H., UNICEF (2007). Iodine Deficiency in Europe: A Continuing Public Health Problem.

[74] Hetzel B.S. (2000). Iodine and neuropsychological development. J. Nutr..

[75] Vejbjerg P., Knudsen N., Perrild H., Laurberg P., Andersen S., Rasmussen L.B., Ovesen L., Jørgensen T. (2009). Estimation of iodine intake from various urinary iodine measurements in population studies. Thyroid.

[76] Richard K., Holland O., Landers K., Vanderlelie J.J., Hofstee P., Cuffe J.S., Perkins A.V. (2017). Review: Effects of maternal micronutrient supplementation on placental function. Placenta.

[77] Vermiglio F., Lo Presti V.P., Moleti M., Sidoti M., Tortorella G., Scaffidi G., Castagna M.G., Mattina F., Violi M.A., Crisa A. (2004). Attention deficit and hyperactivity disorders in the offspring of mothers exposed to mild-moderate iodine deficiency: A possible novel iodine deficiency disorder in developed countries. J. Clin. Endocrinol. Metab..

[78] Sang Z., Wei W., Zhao N., Zhang G., Chen W., Liu H., Shen J., Liu J., Yan Y., Zhang W. (2012). Thyroid dysfunction during late gestation is associated with excessive iodine intake in pregnant women. J. Clin. Endocrinol. Metab..

[79] Connelly K.J., Boston B.A., Pearce E.N., Sesser D., Snyder D., Braverman L.E., Pino S., LaFranchi S.H. (2012). Congenital hypothyroidism caused by excess prenatal maternal iodine ingestion. J. Pediatr..

[80] Besser J.M., Canfield T.J., La Point T.W. (1993). Bioaccumulation of organic and inorganic selenium in a laboratory food chain. Environ. Toxicol. Chem..

[81] Institute of Medicine, Food and Nutrition Board (2000). Dietary Reference Intakes: Vitamin C, Vitamin E, Selenium, and Carotenoids.

[82] Liu X., Zhao Z., Duan B., Hu C., Zhao X., Guo Z. (2015). Effect of applied sulphur on the uptake by wheat of selenium applied as selenite. Plant Soil.

[83] Navarro-Alarcon M., Cabrera-Vique C. (2008). Selenium in food and the human body: A review. Sci. Total Environ..

[84] Dumont E., De Pauw L., Vanhaecke F., Cornelis R. (2006). Speciation of se in Bertholletia excelsa (Brazil nut): A hard nut to crack?. Food Chem..

[85] Dumont E., Vanhaecke F., Cornelis R. (2006). Selenium speciation from food source to metabolites: A critical review. Anal. Bioanal. Chem..

[86] Korotkov K.V., Novoselov S.V., Hatfield D.L., Gladyshev V.N. (2002). Mammalian selenoprotein in which selenocysteine (Sec) incorporation is supported by a new form of Sec insertion sequence element. Mol. Cell. Biol..

[87] Papp L.V., Lu J., Holmgren A., Khanna K.K. (2007). From selenium to selenoproteins: Synthesis, identity, and their role in human health. Antioxid. Redox Signal..

[88] Zimmermann M.B., Köhrle J. (2002). The impact of iron and selenium deficiencies on iodine and thyroid metabolism: Biochemistry and relevance to public health. Thyroid.

[89] Lawrence J.M., Contreras R., Chen W., Sacks D.A. (2008). Trends in the prevalence of preexisting diabetes and gestational diabetes mellitus among a racially/ethnically diverse population of pregnant women, 1999–2005. Diabetes Care.

[90] Dabelea D., Snell-Bergeon J.K., Hartsfield C.L., Bischoff K.J., Hamman R.F., McDuffie R.S. (2005). Increasing prevalence of gestational diabetes mellitus (GDM) over time and by birth cohort: Kaiser permanente of Colorado GDM screening program. Diabetes Care.

[91] Jameson J.L., De Groot L.J. (2015). Endocrinology: Adult and Pediatric.

[92] Chatterjee R., Yeh H.-C., Edelman D., Brancati F. (2011). Potassium and risk of type 2 diabetes. Expert Rev. Endocrinol. Metab..

[93] Vambergue A., Fajardy I. (2011). Consequences of gestational and pregestational diabetes on placental function and birth weight. World J. Diabetes.

[94] Basaki M., Saeb M., Nazifi S., Shamsaei H. (2012). Zinc, copper, iron, and chromium concentrations in young patients with type 2 diabetes mellitus. Biol. Trace Elem. Res..

[95] El-Yazigi A., Hannan N., Raines D.A. (1993). Effect of diabetic state and related disorders on the urinary excretion of magnesium and zinc in patients. Diabetes Research Edinb. Scotl..

[96] Jansen J., Rosenkranz E., Overbeck S., Warmuth S., Mocchegiani E., Giacconi R., Weiskirchen R., Karges W., Rink L. (2012). Disturbed zinc homeostasis in diabetic patients by in vitro and in vivo analysis of insulinomimetic activity of zinc. J. Nutr. Biochem..

[97] MacDonald R.S. (2000). The role of zinc in growth and cell proliferation. J. Nutr..

[98] Zhang C., Rawal S. (2017). Dietary iron intake, iron status, and gestational diabetes. Am. J. Clin. Nutr..

[99] Roberts J.M. (2000). Preeclampsia: What we know and what we do not know. Semin. Perinatol..

[100] McDonald S.D., Han Z., Walsh M.W., Gerstein H.C., Devereaux P.J. (2010). Kidney disease after preeclampsia: A systematic review and meta-analysis. Am. J. Kidney Dis..

[101] Ghulmiyyah L., Sibai B. (2012). Maternal mortality from preeclampsia/eclampsia. Semin. Perinatol..

[102] Morris C.D., Jacobson S.-L., Anand R., Ewell M.G., Hauth J.C., Curet L.B., Catalano P.M., Sibai B.M., Levine R.J. (2001). Nutrient intake and hypertensive disorders of pregnancy: Evidence from a large prospective cohort. Am. J. Obstet. Gynecol..

[103] Hofmeyr G.J., Lawrie T.A., Atallah A.N., Duley L. (2010). Calcium supplementation during pregnancy for preventing hypertensive disorders and related problems. Cochrane Database Syst. Rev..

[104] Jain S., Sharma P., Kulshreshtha S., Mohan G., Singh S. (2010). The role of calcium, magnesium, and zinc in pre-eclampsia. Biol. Trace Elem. Res..

[105] Mistry H.D., Pipkin F.B., Redman C.W., Poston L. (2012). Selenium in reproductive health. Am. J. Obstet. Gynecol..

[106] Ergaz Z., Avgil M., Ornoy A. (2005). Intrauterine growth restriction—Etiology and consequences: What do we know about the human situation and experimental animal models?. Reprod. Toxicol..

[107] Herrera E.A., Alegría R., Farias M., Díaz-López F., Hernández C., Uauy R., Regnault T.R., Casanello P., Krause B.J. (2016). Assessment of in vivo fetal growth and placental vascular function in a novel intrauterine growth restriction model of progressive uterine artery occlusion in guinea pigs. J. Physiol..

[108] Nardozza L.M.M., Araujo Júnior E., Barbosa M.M., Caetano A.C.R., Lee D.J.R., Moron A.F. (2012). Fetal growth restriction: Current knowledge to the general Obs/Gyn. Arch. Gynecol. Obstet..

[109] Figueras F., Gratacos E. (2014). Stage-based approach to the management of fetal growth restriction. Prenat. Diagn..

[110] Henriksen T., Clausen T. (2002). The fetal origins hypothesis: Placental insufficiency and inheritance versus maternal malnutrition in well-nourished populations. Acta Obstet. Gynecol. Scand..

[111] Coan P.M., Vaughan O.R., Sekita Y., Finn S.L., Burton G.J., Constancia M., Fowden A.L. (2010). Adaptations in placental phenotype support fetal growth during undernutrition of pregnant mice. J. Physiol..

[112] Sandovici I., Hoelle K., Angiolini E., Constância M. (2012). Placental adaptations to the maternal–fetal environment: Implications for fetal growth and developmental programming. Reprod. Biomed. Online.

[113] Moore K.L., Persaud T.V.N., Torchia M. (2015). The developing human: Clinically Oriented Embryology.

[114] Wood R.J. (2009). Manganese and birth outcome. Nutr. Rev..

[115] Wang H., Hu Y.-F., Hao J.-H., Chen Y.-H., Su P.-Y., Wang Y., Yu Z., Fu L., Xu Y.-Y., Zhang C. (2015). Maternal zinc deficiency during pregnancy elevates the risks of fetal growth restriction: A population-based birth cohort study. Sci. Rep..

[116] Cao X.-Y., Jiang X.-M., Dou Z.-H., Rakeman M.A., Zhang M.-L., O’Donnell K., Ma T., Amette K., DeLong N., DeLong G.R. (1994). Timing of vulnerability of the brain to iodine deficiency in endemic cretinism. N. Engl. J. Med..

[117] Goldenberg R.L., Culhane J.F., Iams J.D., Romero R. (2008). Epidemiology and causes of preterm birth. Lancet.

[118] Schieve L.A., Tian L.H., Rankin K., Kogan M.D., Yeargin-Allsopp M., Visser S., Rosenberg D. (2016). Population impact of preterm birth and low birth weight on developmental disabilities in US children. Ann. Epidemiol..

[119] Crump C., Sundquist K., Sundquist J. (2016). Adult outcomes of preterm birth. Prev. Med..

[120] Villar J., Abdel-Aleem H., Merialdi M., Mathai M., Ali M.M., Zavaleta N., Purwar M., Hofmeyr J., Campódonico L., Landoulsi S. (2006). World Health Organization randomized trial of calcium supplementation among low calcium intake pregnant women. Am. J. Obstet. Gynecol..

[121] Lieberman E., Ryan K.J., Monson R.R., Schoenbaum S.C. (1988). Association of maternal hematocrit with premature labor. Am. J. Obstet. Gynecol..

[122] Scholl T.O., Hediger M.L., Fischer R.L., Shearer J.W. (1992). Anemia vs iron deficiency: Increased risk of preterm delivery in a prospective study. Am. J. Clin. Nutr..

[123] Dobrzynski W., Szymanski W., Zachara B.A., Trafikowska U., Trafikowska A., Pilecki A. (1998). Decreased selenium concentration in maternal and cord blood in preterm compared with term delivery. Analyst.

[124] Iranpour R., Zandian A., Mohammadizadeh M., Mohammadzadeh A., Balali-Mood M., Hajiheydari M. (2009). Comparison of maternal and umbilical cord blood selenium levels in term and preterm infants. Chin. J. Contemp. Pediatr..

[125] Baxter I. (2010). Ionomics: The functional genomics of elements. Brief. Funct. Genomics.

[126] Zhang P., Georgiou C.A., Brusic V. (2017). Elemental metabolomics. Brief. Bioinforma..

[127] Zhang Y., Shen B. (2017). Trace Elements and Healthcare: A Bioinformatics Perspective. Translational Informatics in Smart Healthcare.

[128] Yu D., Danku J.M., Baxter I., Kim S., Vatamaniuk O.K., Vitek O., Ouzzani M., Salt D.E. (2012). High-resolution genome-wide scan of genes, gene-networks and cellular systems impacting the yeast ionome. BMC Genomics.

[129] Huang X.-Y., Salt D.E. (2016). Plant ionomics: From elemental profiling to environmental adaptation. Mol. Plant.

[130] Ma S., Lee S.-G., Kim E.B., Park T.J., Seluanov A., Gorbunova V., Buffenstein R., Seravalli J., Gladyshev V.N. (2015). Organization of the mammalian ionome according to organ origin, lineage specialization, and longevity. Cell Rep..

[131] Malinouski M., Hasan N.M., Zhang Y., Seravalli J., Lin J., Avanesov A., Lutsenko S., Gladyshev V.N. (2014). Genome-wide RNAi ionomics screen reveals new genes and regulation of human trace element metabolism. Nat. Commun..

[132] Sun L., Yu Y., Huang T., An P., Yu D., Yu Z., Li H., Sheng H., Cai L., Xue J. (2012). Associations between ionomic profile and metabolic abnormalities in human population. PLoS ONE.

[133] Herman M., Golasik M., Piekoszewski W., Walas S., Napierala M., Wyganowska-Swiatkowska M., Kurhanska-Flisykowska A., Wozniak A., Florek E. (2016). Essential and Toxic Metals in Oral Fluid–a Potential Role in the Diagnosis of Periodontal Diseases. Biol. Trace Elem. Res..

